# Identification and Validation of a Major Quantitative Trait Locus for Adult Plant Resistance Against Leaf Rust From the Chinese Wheat Landrace Bai Qimai

**DOI:** 10.3389/fpls.2022.812002

**Published:** 2022-05-18

**Authors:** Zhen Wang, Xu Jiang, Yuzhu Zhang, Ziyi Du, Jing Feng, Wei Quan, Junda Ren, Mingzhe Che, Zhongjun Zhang

**Affiliations:** ^1^Department of Plant Pathology, China Agricultural University, Beijing, China; ^2^School of Agroforestry & Medicine, Open University of China, Beijing, China; ^3^Institute of Plant Protection, Chinese Academy of Agricultural Sciences, Beijing, China; ^4^Beijing Engineering and Technique Research Center for Hybrid Wheat, Beijing Academy of Agricultural and Forestry Sciences, Beijing, China; ^5^Key Laboratory for Northern Urban Agriculture of Ministry of Agriculture and Rural Affairs, Beijing University of Agriculture, Beijing, China

**Keywords:** adult plant resistance, slow rusting, leaf rust, QTL mapping, wheat landrace

## Abstract

Leaf rust caused by *Puccinia triticina* Eriks. (*Pt*) is a common disease of wheat worldwide. The Chinese wheat landrace Bai Qimai (BQM) has shown high resistance to leaf rust for a prolonged period of time; the infected leaves of BQM displayed high infection types (ITs), but they showed low disease severities at the adult plant stage. To find quantitative trait loci (QTL) for resistance to leaf rust, 186 recombinant inbred lines from the cross Nugaines × BQM were phenotyped for leaf rust response in multiple field environments under natural *Pt* infections and genotyped using the 90K wheat single nucleotide polymorphism (SNP) chip and simple sequence repeat (SSR) markers. A total of 2,397 polymorphic markers were used for QTL mapping, and a novel major QTL (*QLr.cau-6DL*) was detected on chromosome 6DL from BQM. The effectiveness of *QLr.cau-6DL* was validated using the three additional wheat populations (RL6058 × BQM, Aikang58 × BQM, and Jimai22 × BQM). *QLr.cau-6DL* could significantly reduce leaf rust severities across all tested environments and different genetic backgrounds, and its resistance was more effective than that of *Lr34*. Moreover, *QLr.cau-6DL* acted synergistically with *Lr34* to confer strong resistance to leaf rust. We believe that *QLr.cau-6DL* should have high potential value in the breeding of wheat cultivars with leaf rust resistance.

## Introduction

The leaf rust of wheat (*Triticum aestivum* L.) caused b*y Puccinia triticina* Eriks. (*Pt*) is a widespread fungal disease that often leads to significant wheat yield losses (Huerta-Espino et al., 2011; Singh et al., 2016). China is the world’s largest wheat producer, and leaf rust epidemics affect approximately 15 million ha in China annually (Liu and Chen, 2012; Zhang L. et al., 2019). Breeding and planting wheat cultivars resistant to leaf rust can reduce fungicide use and is thus an economical and environmentally friendly strategy for managing this disease.

To date, 80 designated leaf rust resistance (Lr) genes and numerous quantitative trait loci (QTL) have been described (Li et al., 2014; McIntosh et al., 2017; Pinto da Silva et al., 2018; Kumar et al., 2021; Zhang et al., 2021). Leaf rust resistance is commonly categorized into two types, namely, all stage resistance (ASR) and adult plant resistance (APR). The ASR *Lr* genes/QTL are generally only effective against a subset of *Pt* races in the gene-for-gene manner (Flor, 1971), which is pathologically characterized by hypersensitive response with low infection type (IT). The race-specific resistance genes usually confer the high levels of resistance against avirulent pathotypes, but some of them have lost their effectiveness due to the continuous evolution of virulent pathotypes in commercial wheat fields, which is exemplified by *Lr16* and *Lr26* in China (Liu and Chen, 2012) and *Lr21*, *Lr24*, *Lr37*, and *Lr39* in the United States (Kolmer et al., 2018).

In contrast, APR genes can be race-specific or non-race-specific. Non-race-specific APR genes reduce disease severity or condition partial resistance by limiting disease development, such as prolonging the latent period, restricting the lesion size, and reducing spore production. The pleiotropic APR genes *Lr34*/*Yr18*/*Sr57* and *Lr67*/*Yr46*/*Sr55* are reported to be durable and confer slow rusting resistance to all current races of multiple pathogens, even though the wheat lines carrying *Lr34* or *Lr67* often display susceptible ITs (Krattinger et al., 2009; Moore et al., 2015). The cloned *Lr34* and *Lr67* encode a predicted ATP-binding cassette transporter and a predicted hexose transporter, respectively (Krattinger et al., 2009; Moore et al., 2015), which are distinct from the race-specific resistance genes that commonly encode nucleotide-binding leucine-rich repeat domains (Dangl et al., 2013). Other well-characterized leaf rust APR genes include *Lr46*, *Lr68*, *Lr74*, *Lr75*, *Lr77*, and *Lr78*, which are located on chromosomes 1BL, 7BL, 3BS, 1BS, 3BL, and 5DL (Pinto da Silva et al., 2018 and references therein). These genes differ in effect size (i.e., the phenotypic variance explained by a single QTL) and may provide insufficient protection under severe *Pt* epidemics when deployed individually. Thus, effective ASR and APR genes need to be combined to ensure high-level resistance by marker-assisted selection (MAS; Lagudah, 2011), and new sources of resistance need to be identified to enhance the genetic diversity of leaf rust resistance.

Wheat landraces are the valuable sources of resistance to diseases. The winter wheat line Bai Qimai (BQM), a Chinese landrace without available pedigree information, was cultivated widely in the southern Gansu Province of China from the 1860s to 1968 (Zhang, 1995), and there are historical records of leaf rust resistance in BQM. The objectives of this study were to (1) detect QTL associated with the resistance in BQM, (2) evaluate the effect size of the detected QTL with *Lr34* as a baseline, and (3) validate the effectiveness of the detected QTL across different genetic backgrounds and tested environments.

## Materials and Methods

### Wheat Materials

The wheat cross Nugaines (NG) × Bai Qimai (BQM) was used to map QTL with BQM as pollen donor. NG (CItr13968) is an American wheat cultivar (Vogel and Peterson, 1974) that is susceptible to leaf rust. A recombinant inbred line (RIL) population of the cross was developed to the F_6_ generation using single seed descent method. From the F_6_ plants of each RIL, one spike was randomly sampled to grow F_7_ spike-row plants. One seed from the spike-row was used as the representative of F_8_ generation, and the remaining seeds were bulked to grow F_6:8_ plants. Each RIL traces back to a single F_6_ plant. Likewise, the representative seed and bulked seeds of the subsequent generations were developed. A total of 186 F_6_-derived RILs were used for phenotyping the response to leaf rust in the fields of 2015 (F_8:10_), 2016 (F_9:11_), and 2017 (F_10:12_). DNA from the F_9_ generation of each RIL was used for single nucleotide polymorphism (SNP) genotyping. The wheat line Mingxian169 (MX), which is highly susceptible to leaf rust (Du et al., 2015), was used as a susceptible check throughout the experiments.

Three additional wheat populations were utilized to validate the detected QTL, namely, 80 F_2:3_ families of RL6058 × BQM, 10 BC_3_F_2:3_ families of Aikang58 (AK58) × BQM, and 10 BC_3_F_2:3_ families of Jimai22 (JM22) × BQM (AK58 and JM22 as recurrent parents) (for more information, refer to Supplementary Figure 1). RL6058 is a Thatcher backcross line with *Lr34* that shows APR to leaf rust (Krattinger et al., 2009). AK58 and JM22 are elite Chinese wheat commercial cultivars that are susceptible to leaf rust.

### Evaluation of Bai Qimai for Leaf Rust Reaction

Under controlled greenhouse conditions, the seedlings and adult plants of BQM together with other wheat genotypes (NG, RL6058, AK58, JM22, and MX) were assessed with the *Pt* races THTT and FHTR, respectively, which collectively have a wide virulence spectrum (Li et al., 2010). For seedling tests, 3–5 plants of each line were grown in 9-cm-diameter pots; for adult plant tests, plants from the first leaf stage were vernalized at 4°C with a 16-h photoperiod for 30 days and subsequently transplanted to larger 23-cm diameter pots. Seedlings at the two leaf stage and adult plants at the booting stage were uniformly dust-inoculated with urediniospores of the races THTT and FHTR, respectively. Inoculated plants were incubated in a dew chamber (100% relative humidity) at 18°C overnight (18 h) and were then returned to the greenhouse at 20 ± 2°C with 16 h of light (22,000 lx) daily. Briefly, 12 to 15 days after inoculation, IT was recorded using a standard 0–4 scale (Long and Kolmer, 1989). Leaf rust severity was measured as the percentage of leaf area infected and was only scored for adult plants using the modified Cobb scale (Peterson et al., 1948). To confirm phenotypes, the tests were repeated three times.

In the field nursery in Wushan County, Gansu Province, China, the leaf rust response of BQM was tested under natural *Pt* infections from 1987 to 2020. BQM was planted in three blocks with MX as the susceptible check, and an individual plot consisted of a 1-m long row. Leaf rust severity was scored on five flag leaves from each plot, and the mean value was calculated by averaging leaf rust severity scores over three replicates within each year.

### Field Phenotyping of the Mapping Population

The 186 NG × BQM RILs and their parents were phenotyped under natural *Pt* infections in three autumn-sown wheat crop seasons (2014–2015, 2015–2016, and 2016–2017; abbreviated as 2015, 2016, and 2017, respectively) and two locations, i.e., Southern Gansu Province (Wushan county; 34°42′15″N, 104°40’08″E; elevation of 1950 m; annual precipitation 538.4 mm) and Shandong Province (Tai’an district; 36°18′09″N, 117°13′05″E; 90 m; 750.4 mm). The autumn-sown wheat crop season lasts from mid-September to late July in Gansu and from early October to June in Shandong. The plant materials were arranged in a randomized complete block design with three replications per location. Each entry was planted as a single row plot of 1 m long with 25 cm between rows, and approximately 40 seeds of RILs were sown in each plot. The parental lines and susceptible check were included after every 60 RIL rows. Two rows of MX were planted around each field block as spreaders to mediate the uniformity of the leaf rust epidemics throughout the trials. The tested plants were covered with plastic films during winter months to help the plants overwinter. The RILs and parents were evaluated for leaf rust severity and response on five leaves collected from different parts of a row. Recording was done three times when the flag leaves of NG and MX showed disease severities of 10–30%, 50–60%, and 80–90%, respectively, within the period corresponding to wheat growth stages from 55 to 77 in the scale of Zadoks et al. (1974). The area under the disease progress curve (AUDPC) was calculated based on three times recordings for each plot entry according to Wang et al. (2019). QTL analysis was based on the mean AUDPC calculated by averaging the three replicates within an individual environment.

### Phenotyping of the Validating Population

To examine QTL effect and MAS effectiveness, the selected F_2:3_ and BC_3_F_2:3_ families from the three additional wheat populations were assessed for leaf rust severities under greenhouse conditions in 2018. Each population was tested in a separate trial, and each trial had three replicates arranged in a randomized complete block design. For RL6058 × BQM, there were four plots per replicate, and each plot was sown with 20 F_2:3_ families (10 plants within each F_2:3_ family) from one of the four groups (i.e., *QLr.cau-6DL* + *Lr34*, *QLr.cau-6DL*, *Lr34*, and None). For AK58 × BQM or JM22 × BQM, an individual replicate had two plots sown with the R-group and S-group, and each group contained 5 BC_3_F_2:3_ families (10 plants within each BC_3_F_2:3_ family). Sowing was conducted in early October of the previous year. From late November to early February, greenhouse heating was turned off, and the seedlings were conditioned with temperatures of −3°C (the lowest, night) to 7°C (the highest, day) to ensure vernalization. Afterward, the greenhouse was maintained at 18°C (the lowest, night) to 25°C (the highest, day) daily with a 16-h light/8-h dark photoperiod. In early March, the plants were spray-inoculated with a mixed urediniospores of *Pt* races (THTT:FHTR = 1:1) suspended in water containing 0.03% Tween 20 (approximately 80,000 urediniospores/ml). Inoculated plants were covered with plastic films and incubated overnight (18 h) at 18 ± 1°C. The films were then removed and the greenhouse was operated at 18 to 25°C daily with the 16-h light/8-h dark photoperiod. The plants were frequently misted after sunset to facilitate re-infection. The disease severity was measured when the MX flag leaves showed the severities of 80–90%. For each F_2:3_ or BC_3_F_2:3_ family, the mean value was calculated by averaging the 10 plants within each family. The leaf rust severities of 80 RL6058 × BQM F_2:3_ families were evaluated under natural *Pt* infections in the field nursery in the Southern Gansu Province in 2018. The disease severity assessment and AUDPC calculation were performed in the same ways described above in the field trials.

### Statistical Analysis

All analyses were performed in SAS version 9.4 (SAS Institute Inc., Cary, NC, United States). Analysis of variance (ANOVA) was conducted based on AUDPC values across six field trials for NG × BQM. A linear model was fitted using PROC GLM: *P*_*ijk*_ = μ + *G*_*i*_ + *E*_*j*_ + *E*_*j*_(*R*_*k*_) + *G_*i*_* × *E_*j*_* + *e*_*ijk*_, where *P*_*ijk*_ is the phenotypic value, μ is the population mean, *G*_*i*_ is the effect of the ith genotype (RIL), *E*_*j*_ is the effect of the jth environment, *E*_*j*_(*R*_*k*_) is the effect of the kth replicate within the jth environment, *G_*i*_* × *E*_*j*_ is the ijth effect of the genotype-by-environment interaction, and *e*_*ijk*_ is the residual. Broad-sense heritability (*H*^2^) was estimated using PROC VARCOMP (method = REML): *H*^2^ = σ*^2^_*G*_*/(σ*^2^_*G*_* + σ^2^_*error*/_r), where σ*^2^_*G*_* denotes the genotypic (RIL) variance, σ^2^_*error*_ is the error variance, and r is the number of replicates (Holland et al., 2003). Correlation coefficients between different trials were estimated applying PROC CORR (Pearson’s). For RL6058 × BQM, AK58 × BQM and JM22 × BQM, the mean value averaged the plants within each group was considered as one experimental unit. ANOVA was performed using PROC GLM by fitting the model *P_*ik*_* = μ + *G_*i*_* + *R_*k*_* + *e*_*ik*_. The comparison of phenotypic values between groups were performed according to Fisher’s least significant difference test at α = 0.0001.

### Genotyping, Map Construction, and Quantitative Trait Loci Analysis

Conventional bulked segregant analysis (BSA) was used to screen for markers linked to the resistant QTL. Because of the contrasting leaf rust resistance phenotypes in the 186 NG × BQM RILs, two DNA bulks containing 20 extremely resistant or susceptible RILs were screened using more than 2,300 simple sequence repeat (SSR) markers spanning 21 wheat chromosomes to identify those for which the two bulks were polymorphic. The detected polymorphic markers were used to genotype the above selected 40 RILs, and the genotypes and phenotypes were then used to perform marker–trait association analysis using the single marker analysis method in Windows QTL Cartographer 2.5 (Wang et al., 2010).

For genome-wide linkage mapping, the 186 NG × BQM RILs and their parents were genotyped using the 90K wheat SNP chip (Wang et al., 2014) by CapitalBio Technology (Beijing, China).^1^ SNP calling and clustering were performed with GenomeStudio V1.9.4 software (Illumina).^2^ Redundant markers that showed complete linkage and markers missing more than 10% or with significant (*p* < 0.01) segregation distortion were removed using the BIN function in QTL ICIMapping V4.3 (Li et al., 2007).^3^ Genetic maps were constructed using the software Joinmap 4.0 (Stam, 1993)^4^ and the MSTmap program (Wu et al., 2008)^5^ with the Kosambi function. Each linkage group was assigned to a specific chromosome by referring to the 90K SNP consensus map (Wang et al., 2014).

Quantitative trait loci analysis was performed using the Composite Interval Mapping method in Windows QTL Cartographer 2.5 (Wang et al., 2010). The threshold logarithm of odds (LOD) score was calculated by running the permutation program with 3,000 replications at a type I error rate of α = 0.05; for simplicity, the highest threshold LOD (2.9) value was used as a uniform threshold for all tested environments. Only QTL that exceeded the threshold LOD value in at least two environments were described here. The determination coefficient [i.e., phenotypic variation explained (PVE)] was used to measure the effect size of QTL.

To obtain the physical positions of QTL, the SNP and SSR marker probes were aligned with the International Wheat Genome Sequencing Consortium (IWGSC) RefSeq v2.1 using IWGSC BLAST. For previously reported QTL, the closest flanking markers were used to generate confidence intervals (Maccaferri et al., 2015).

## Results

### Characterization of the Bai Qimai Resistance

**FIGURE 1:**
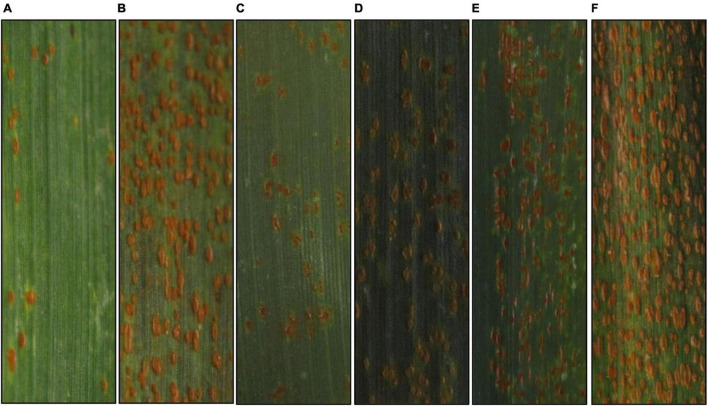
Typical symptoms of the parental and control wheat lines infected by *Puccinia triticina* race THTT under greenhouse conditions. **(A–F)** Denote Bai Qimai (BQM), Nugaines (NG), RL6058, Aikang58 (AK58), Jimai22 (JM22), and Mingxian169 (MX) at the adult plant stage, respectively.

In the greenhouse tests, BQM showed susceptibility with ITs 3 + to the *Pt* races THTT and FHTR at both the seedling and adult plant stages; however, disease severities were low (<20%) at the adult plant stage. For comparison, the susceptible parent NG and check MX consistently showed high susceptibility (ITs 4, severities >80%) to both races at the same growth stages (Figure 1 and Table 1). In the field, we observed BQM plants infected with naturally occurring *Pt* populations with MX as control from 1987 to 2020, showing that the flag leaves of BQM displayed leaf rust severities ranging from 10 to 25%, and MX showed severities of 85–100% (Supplementary Table 1). These results indicated that BQM confers slow rusting resistance to leaf rust at the adult plant stage.

**TABLE 1:** Infection type (IT)^a^ and disease severity (%)^b^ of the parental wheat lines and check under greenhouse conditions and in the fields.

	Greenhouse^c^				Field^c^
	Seedling		Adult plant		Adult plant
Wheat line	THTT	FHTR	THTT	FHTR	Natural infections
Bai Qimai	4	4	3 + /17.4	3 + /16.7	3 + /19.7
Nugaines	4	4	4/85.3	4/86.7	4/89.3
RL6058	4	4	3 + /30.7	3 + /28.7	3 + /35.2
Aikang58	4	4	4/76.7	4/75.4	–
Jimai22	4	4	4/78.7	4/79.3	–
Mingxian 169	4	4	4/96.7	4/96.0	4/88.9

*^a^Infection types are based on a 0–4 scale (Long and Kolmer, 1989), where 3 = moderate size uredinia without chlorosis or necrosis, 4 = large size uredinia without chlorosis or necrosis, and + = uredinia somewhat larger than normal for infection type. ^b^Disease severity was scored on adult plants using the modified Cobb scale (Peterson et al., 1948), and the mean values were calculated by averaging over plants with repeated trials. ^c^In the greenhouse trial, the tested plants were inoculated with the Puccinia triticina races THTT and FHTR, while in the field trial, plants were infected by the naturally occurring P. trticina population.*

### Leaf Rust Phenotypes of the Recombinant Inbred Line Population

**FIGURE 2:**
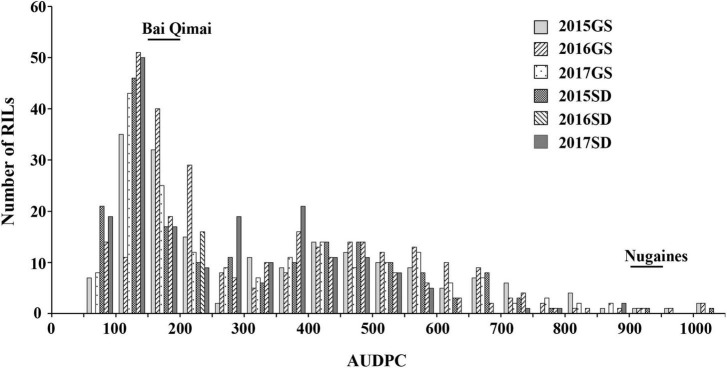
Frequency distribution of leaf rust severities (measured in AUDPC) for the 186 recombinant inbred lines from NG × BQM in field trials under natural infections of *Puccinia triticina during* 2015–2017 in Gansu (2015GS, 2016GS, and 2017GS) and Shandong (2015SD, 2016SD, and 2017SD).

The NG × BQM RIL population and their parental lines were phenotyped under natural *Pt* infections in the six field environments. IT values for the 186 RILs varied little (IT 3–4 on all RILs except for six with IT 2–3); therefore, only severity values were analyzed. The disease severity of NG and MX was greater than 80% (Table 2), indicating that the disease pressure was sufficiently high for evaluating resistance. To improve the power of QTL detection, the severity values were measured in AUDPC (Supplementary Table 2). The AUDPC distributions among the 186 RILs in all six environments were continuous, and their patterns were similar (Figure 2), indicating that leaf rust resistance was quantitatively inherited. ANOVA showed that genetic and environmental effects and their interaction were significant (*p* < 0.0001) (Table 3); *H*^2^ was estimated to be 0.96, suggesting the presence of a major QTL. The correlation coefficients ranged from 0.75 to 0.90 and were significant (*p* < 0.0001) (Table 4), indicating a high similarity in the rank order of leaf rust resistance of RILs across the six field trials.

**TABLE 2:** Leaf rust severities of the parental wheat lines Bai Qimai (BQM) and Nugaines (NG), the check Mingxian169 (MX), and the recombinant inbred line (RIL) population of NG × BQM in field trials under natural infections of *Puccinia triticina* during 2015–2017 in Gansu (2015GS, 2016GS, and 2017GS) and Shandong (2015SD, 2016SD, and 2017SD).

	Parental lines		RIL population			Check
Test environment	BQM	NG	Mean	Min	Max	MX
2015GS	16.7 (165)^a^	90.3 (972)	33.1 (325)	2.4 (29)	93.3 (1076)	90.2 (1045)
2016GS	17.1 (170)	91.2 (988)	34.5 (356)	5.0 (63)	99.0 (1283)	91.1 (1163)
2017GS	17.8 (169)	90.8 (957)	33.1 (306)	2.4 (27)	96.7 (1185)	90.5 (1055)
2015SD	19.5 (158)	90.0 (924)	30.1 (280)	2.3 (26)	96.7 (1052)	88.7 (1048)
2016SD	22.7 (181)	89.5 (904)	29.9 (263)	2.6 (27)	93.3 (1091)	89.6 (1036)
2017SD	20.3 (155)	83.7 (904)	27.7 (242)	1.5 (17)	85.1 (1043)	84.7 (1014)

*^a^Disease severity was indicated by percentage of infected leaf area (%) and the area under the disease progress curve (AUDPC; in parenthesis). AUDPC was calculated based on the data in Supplementary Table 2.*

**TABLE 3:** Analysis of variance (ANOVA) of leaf rust severities (AUDPC) for the 186 recombinant inbred lines (RILs) of NG × BQM in different field environments.

Source	*df*	Mean square	*F*-value	*P*-value	*H* ^2^
RIL	185	608130.8	82.47	<0.0001	0.96
Environment	5	986828.3	133.83	<0.0001	
RIL × Environment	925	24586.7	3.33	<0.0001	
Replicate (environment)	2	18506.5	2.51	0.0028	
Error	2214	7373.7			

**TABLE 4:** Correlation coefficients for leaf rust severities (AUDPC) of the 186 RILs of NG × BQM among the six field tests during 2015–2017 in Gansu (2015GS, 2016GS, and 2017GS) and Shandong (2015SD, 2016SD, and 2017SD).

Test	2016GS	2017GS	2015SD	2016SD	2017SD
2015GS	0.81****^a^	0.81****	0.81****	0.79****	0.85****
2016GS		0.77****	0.80****	0.81****	0.90****
2017GS			0.75****	0.75****	0.82****
2015SD				0.76****	0.85****
2016SD					0.85****

^a^****significant at P < 0.0001.

### Map Construction and Quantitative Trait Loci Detection

A total of 2,344 SSR markers on 21 wheat chromosomes were screened using the two DNA bulks and parents, of which 78 SSR markers were identified to be polymorphic and were used to genotype the 40 RILs of the contrasting bulks. Marker–trait association analysis based on the genotypic and phenotypic data revealed that cfd188 was the most significant (*p* < 0.0001) marker, and it was consistently associated with leaf rust resistance across all trials. According to the consensus SSR map of Somers et al. (2004) and the deletion bin physical map of Sourdille et al. (2004), cfd188 is located on the long arm of chromosome 6D, suggesting that the resistance QTL was located in this region.

After the 90K SNP assays, 8,047 SNP markers were polymorphic with known chromosome locations for the 186 NG × BQM RILs and their parents. The remaining markers were excluded from subsequent analyses due to monomorphy, high frequencies of missing data (≥10%), or distorted marker segregation (α = 0.01). Following QTL location to chromosomes 6D, SSR markers on the target chromosomes were screened for polymorphism between the parents, and the polymorphic markers were used to genotype the 186 RILs. After removing redundant markers, the final 2,374 SNP markers and 23 SSR markers were used to construct the genetic bin map, which covered 3,248.9 cM with an average interval of approximately 1.4 cM between adjacent markers (Supplementary Table 3). These markers were assigned to 22 linkage groups corresponding to the 21 chromosomes, with 2D represented by two linkage groups and other chromosomes each represented by one (Supplementary Table 3).

**FIGURE 3:**
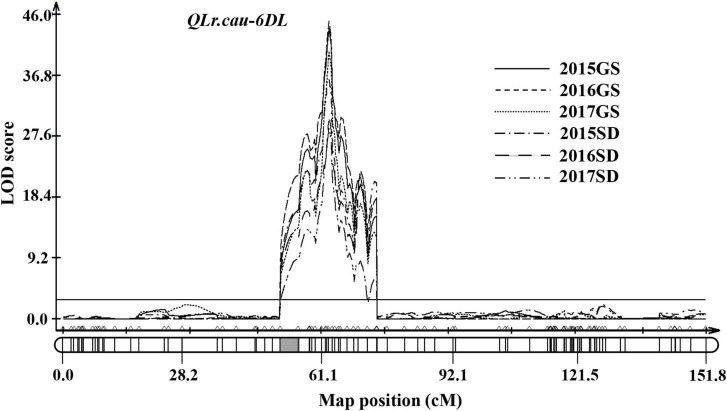
The logarithm of odds (LOD) curves of *QLr.cau-6DL* detected on chromosome 6D of NG × BQ using leaf rust severities (AUDPC) in field trials under natural infections of *Puccinia triticina* during 2015–2017 in Gansu (2015GS, 2016GS, and 2017GS) and Shandong (2015SD, 2016SD, and 2017SD). The horizontal line indicates the threshold LOD at 2.9. The gray box indicates the position of the centromere inferred by aligning marker sequences to the chromosome survey sequence.

In total, 186 RILs were scanned genome-wide with the 2,397 markers to detect the chromosome regions associated with the AUDPC values for each of the six field experiments. A major QTL was detected on chromosome 6DL and designated as *QLr.cau-6DL* (Figure 3). The LOD peaks of *QLr.cau-6DL* were located between the marker cfd188 and IWB55857 within a 0.9-cM interval. The alignment of sequences of the flanking markers with IWGSC RefSeq v2.1 (International Wheat Genome Sequencing Consortium [IWGSC], 2018) indicated that *QLr.cau-6DL* was located in the chromosome 6D genome interval 259.41–313.75 Mb. LOD peak values, which ranged from 28.9 to 44.8, were significantly larger than the LOD threshold (2.9) in the six field experiments. The parent BQM contributed to the APR resistance at *QLr.cau-6DL*, which explained from 34 to 64% of the phenotypic variance (Table 5).

**TABLE 5:** Quantitative trait loci (QTL) associated with adult plant resistance to leaf rust detected on the basis of disease severities (AUDPC) in the NG × BQM population tested under natural infections of *Puccinia triticina* during 2015–2017 in Gansu (2015GS, 2016GS, and 2017GS) and Shandong (2015SD, 2016SD, and 2017SD).

Population/QTL	Test	Chr. arm	Adjacent marker (physical position, Mb)^a^	LOD^b^	PVE (%)^c^	Donor^d^
**Analysis on the complete set of 186 RILs/**						
*QLr.cau*-*6DL*	2015GS	6DL	*cfd188* (259.41) *IWB55857* (313.75)	43.4	59	BQM
	2016GS	6DL	*cfd188* (259.41) *IWB55857* (313.75)	44.8	64	BQM
	2017GS	6DL	*cfd188* (259.41) *IWB55857* (313.75)	40.3	48	BQM
	2015SD	6DL	*cfd188* (259.41) *IWB55857* (313.75)	36.3	45	BQM
	2016SD	6DL	*cfd188* (259.41) *IWB55857* (313.75)	30.7	46	BQM
	2017SD	6DL	*cfd188* (259.41) *IWB55857* (313.75)	28.9	34	BQM
**Analysis on the subset of 96 RILs, with negative states for cfd188/**						
*QLr.cau*-*5BL*	2015GS	5BL	*IWB12416* (559.44)	4.6	12	BQM
	2017SD	5BL	*IWB12416* (559.44)	4.8	13	BQM
*QLr.cau*-*5DL*	2015GS	5DL	*IWB10111* (529.73)	2.9	9	NG
	2017GS	5DL	*IWB10111* (529.73)	4.4	12	NG

*^a^Physical position was inferred by aligning the adjacent marker sequence with IWGSC RefSeq v2.1. ^b^LOD = logarithm of odds. ^c^PVE = the phenotypic variation explained by QTL. ^d^Donor = the parental line with the resistance allele.*

Two additional QTL were detected on the chromosomes 5BL and 5DL on the basis of the 96 RILs, which were selected from the 186 NG × BQM RILs based on negative states for the marker cfd188 (i.e., with susceptible alleles at *QLr.cau-6DL*). These two QTL were designated as *QLr.cau-5BL* and *QLr.cau-5DL*, and resistant alleles were derived from BQM at *QLr.cau-5BL* and NG at *QLr.cau-5DL*. They showed limited effect sizes (PVE = 9–13%) and were effective in only two of the six field experiments (Table 5). Thus, only the major QTL *QLr.cau-6DL* was further examined in this paper.

### Effect of *QLr.cau-6DL* on Leaf Rust Resistance

**FIGURE 4:**
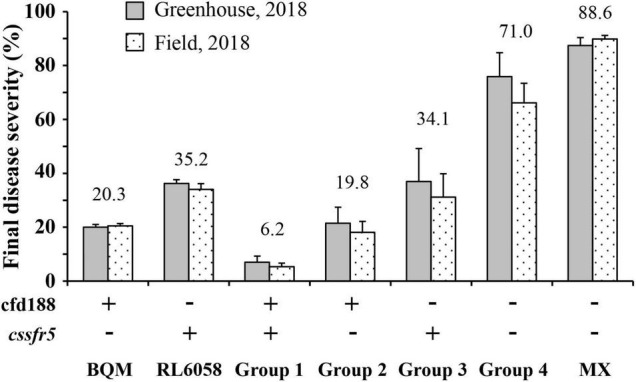
Final disease severities on the RL6058 × BQM F_2:3_ family groups containing different quantitative trait loci (QTL) combinations. Group 1 plants were positive for both cfd188 and *cssfr5* (+ , +), Group 2 plants were positive for cfd188 and negative for *cssfr5* (+ , -), Group 3 plants were negative for cfd188 and positive for *cssfr5* (-, +), and Group 4 plants were negative for both cfd188 and *cssfr5* (-, -). Tests were conducted under greenhouse conditions and in the field of Southern Gansu Province in 2018.

**FIGURE 5:**
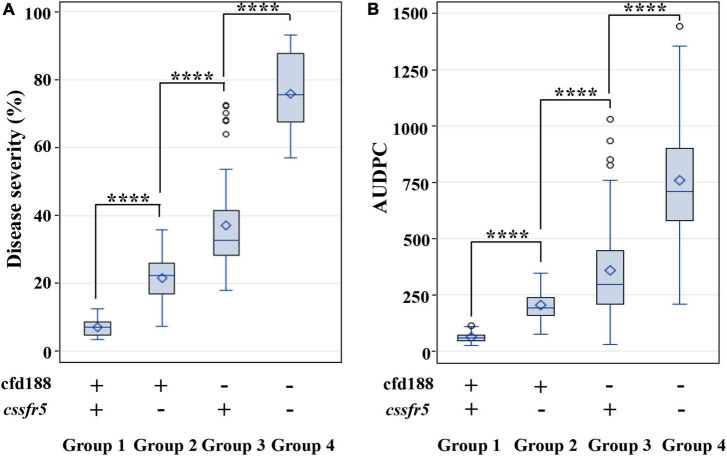
Boxplots showing the effects of the F_2:3_ family groups on leaf rust severities (or AUDPC) in the RL6058 × BQM cross. Refer to the caption of panel Figure 4 for the F_2:3_ family grouping. Tests were conducted in 2018 under greenhouse conditions **(A)** and in the field of Souther Gansu Province **(B)**. **** indicates significant difference at *p* < 0.0001 based on Fisher’s least significant difference test. Within each box, the small diamond and the horizontal line indicate the mean and median area under the disease progress curve (AUDPC), respectively. The top and bottom edges of a box illustrate the 75th and 25th percentiles, respectively. Whiskers (vertical lines outside a box) extend to the extreme data points, and small circles denote outliers.

The *QLr.cau-6DL* effect was validated using the three additional wheat crosses: RL6058 × BQM, AK58 × BQM, and JM22 × BQM (Supplementary Figure 1). RL6058 has leaf rust resistance gene *Lr34*. The marker adjacent to *QLr.cau-6DL*, cfd188, and the *Lr34*-specific marker *cssfr5* were used to select four groups of QTL combination from the RL6058 × BQM cross. The F_2_ seedlings of Group 1 were positive for both cfd188 and *cssfr5* (representing *QLr.cau-6DL* + *Lr34*), the F_2_ seedlings of Group 2 were positive for cfd188 and negative for *cssfr5* (*QLr.cau-6DL*), the F_2_ seedlings of Group 3 were negative for cfd188 and positive for *cssfr5* (*Lr34*), and the F_2_ seedlings of Group 4 were negative for both cfd188 and *cssfr5* (None). For each group, 20 F_2_ seedlings were sampled and advanced to F_2:3_ families, and the F_2:3_ families were evaluated for leaf rust severities at the adult plant stage. Each group plants displayed different levels of leaf rust resistance (Supplementary Table 4), indicating that selection for *QLr.cau-6DL* based on cfd188 was as effective as selection for *Lr34* based on *cssfr5*. *QLr.cau-6DL* and *Lr34* could reduce final leaf rust severity by 51.2% and 36.9% on average, respectively, and their effectiveness was visually identical to the resistant parents (Figure 4). The *QLr.cau-6DL* + *Lr34* combination reduced the final leaf rust severity by 64.8% cumulatively. Significant differences (*p* < 0.0001) were observed among these groups (Table 6). Disease severities or AUDPC values were the lowest for Group 1, followed by Group 2, Group 3, and Group 4 (Figure 5). These results suggested that the plants with both *QLr.cau-6DL* and *Lr34* had the highest resistance level, and that the plants containing *QLr.cau-6DL* had a higher level of resistance than those containing *Lr34*.

**TABLE 6:** Analysis of variance of leaf rust severities for the selected F_2:3_ family groups from RL6058 × BQM under greenhouse conditions and in the Gansu field in 2018, and the selected BC_3_F_2:3_ family groups from AK58 × BQM and JM22 × BQM under greenhouse conditions in 2018.

Population/Test^a^	Source	*df*	Mean square	*F*-value	*P*-value
RL6058 × BQM/	QTL group^b^	3	52801.2	589.91	<0.0001
Greenhouse, 2018	Replicate	2	42.4	0.47	0.6233
	Error	234	89.5		
RL6058 × BQM/	QTL group^b^	3	5408930.9	171.37	<0.0001
Field, Gansu, 2018	Replicate	2	44743.1	1.42	0.2444
	Error	234	31563.3		
AK58 × BQM/	QTL group^c^	1	24946.6	534.73	<0.0001
Greenhouse, 2018	Replicate	2	182.9	3.92	0.0325
	Error	26	46.7		
JM22 × BQM/	QTL group^c^	1	25137.3	532.29	<0.0001
Greenhouse, 2018	Replicate	2	167.1	3.54	0.0437
	Error	26	47.2		

*^a^Analysis of variance was conducted based on the final disease severity in the greenhouse trials and AUDPC values in the field trial. ^b^The groups were selected based on the positive or negative states for the markers cfd188 and cssfr5, respectively. The markers cfd188 and cssfr5 represent QLr.cau-6DL and Lr34, respectively. ^c^The groups were selected based on the positive or negative states for the marker cfd188.*

**FIGURE 6:**
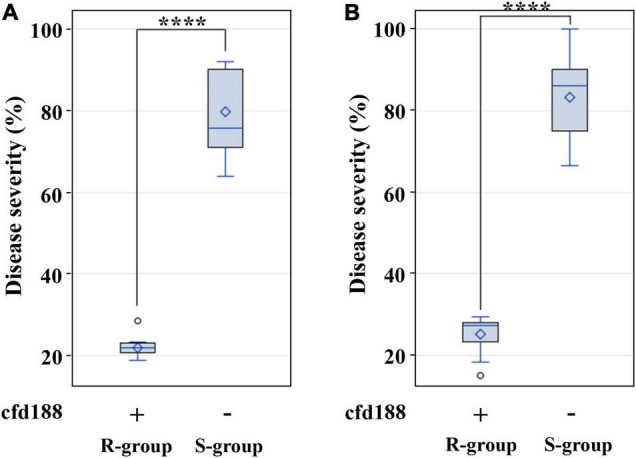
Boxplots showing the effect of *QLr.cau-6DL* on leaf rust severities in the BC_3_F_2:3_ family groups from Aikang58 (AK58) × BQM **(A)** and Jimai22 (JM22) × BQM **(B)**. R-group plants were positive for cfd188 (+), and S-group plants were negative for cfd188 (-). Tests were conducted under greenhouse conditions in 2018. **** indicates significant difference at *p* < 0.0001 based on Fisher’s least significant difference test. Refer to the caption of panel Figure 5 for descriptions of box.

From the AK58 × BQM cross, two BC_3_F_2:3_ family groups were selected for *QLr.cau-6DL* based on cfd188, i.e., one group with positive states for cfd188 (R-group) and the other group with negative states for cfd188 (S-group). Disease severities were significantly (*p* < 0.0001) lower for R-group BC_3_F_2:3_ plants than S-group plants (Table 6, Figure 6A and Supplementary Table 5). There was a significant difference (*p* < 0.0001) between these two groups in the JM22 × BQM cross (Table 6, Figure 6B and Supplementary Table 5). Thus, *QLr.cau-6DL* was well effective in the populations AK58 × BQM and JM22 × BQM.

## Discussion

The Chinese wheat landrace BQM consistently showed high resistance to naturally occurring *Pt* populations with severity of 10–25% at the adult plant stage in the Southern Gansu Province from 1987 to 2020. In contrast, the susceptible check MX always showed high susceptibility (severity 85–100%) in the same nursery (Supplementary Table 1). In this study, a major QTL *QLr.cau-6DL* was mapped to chromosome 6DL, with the resistant allele contributed by BQM. One *Lr* gene (*Lr38*) and 15 QTL have been previously reported to be located on chromosome 6D. The *Lr38* gene confers hypersensitive resistance to leaf rust and was introgressed into *T. aestivum* from *Agropyron intermedium* (Mebrate et al., 2008), indicating that *Lr38* is genetically different from *QLr.cau-6DL*. The 15 QTL are diverse with respect to the resistance component, effectiveness magnitude, and effect consistency.

Five QTL (*QLr.cimmyt-6DS*, *QLr.B22-6D*, *QLrs.B22-6D*, *QLr.hebau-6DS*, and *QLr.cdl-6D*; Supplementary Table 6) were mapped using biparental populations, and the other ten were detected using wheat panels for association mapping (AM) or genome-wide association study (GWAS). *QLr.cimmyt-6DS* is located on chromosome 6DS and reduces disease severity with a PVE value of 9.0% from Indian spring wheat (Sukhwinder-Singh et al., 2012). *QLr.B22-6D* and *QLrs.B22-6D* originate from the synthetic wheat accession Syn022L. The former shows field resistance to leaf rust and functions in only some environments, and the latter is expressed at the seedling stage and results in a 21% reduction in disease severity (Naz et al., 2008). *QLr.hebau-6DS* from Thatcher is detected in only one of the three environments with a PVE of 6.3% (Zhang P. et al., 2019). *QLr.cdl-6D* from the Uruguayan cultivar Americano-44 has PVE values up to 23% in field tests but is ineffective for conferring resistance in greenhouse tests (Kolmer et al., 2018). The ten AM or GWAS QTL (*QLr.IWA2476*, *QLr.wPt-1695*, *QLr.IWB33802*, *QLr.IWB18070*, *QLr.IWB9015*, *QLr.IWB505*, *QLr.IWB10505*, *QLr.IWA619*, *QLr.IWA7616*, and *QLr.IWA6181*; Supplementary Table 6) were mapped using diverse wheat panels involving spring wheat varieties and winter-habit hexaploid wheat landraces. *QLr.IWA2476* conditions field resistance only at the adult plant stage (Turner et al., 2017). *QLr.wPt-1695* is associated with low leaf rust responses in one of three crop seasons (Bansal et al., 2013). *QLr.IWB33802*, *QLr.IWB18070*, *QLr.IWB9015*, *QLr.IWB505*, and *QLr.IWB10505* induce field resistance with PVE values ranging from 18 to 48% (Leonova et al., 2020). *QLr.IWA619* and *QLr.IWA7616* confer resistance to *Pt* races TBDJ and TDBJ, respectively, at the seedling stage (Kertho et al., 2015). *QLr.IWA6181* is associated with low disease severity and host response at the adult plant stage (Kankwatsa et al., 2017).

We inferred the physical positions of the 15 QTL by aligning their resistance-associated marker sequences with the IWGSC RefSeq v2.1 (International Wheat Genome Sequencing Consortium [IWGSC], 2018). All these QTL (except *QLr.cdl-6D* and *QLr.IWB33802*) were separated from *QLr.cau-6DL* by at least 50 Mb (Supplementary Table 6). *QLr.cdl-6D* reduced the leaf rust severity in field plot tests, but it was shown to be ineffective in greenhouse tests. Moreover, *QLr.cdl-6D* originated from the Uruguayan cultivar Americano-44 (PI-191937)^6^ (Kolmer et al., 2018). Leonova et al. (2020) found *QLr.IWB33802* from the 100 Russian varieties of spring wheat through genome-wide association mapping, while they did not specify the accession that harbors the resistance allele at *QLr.IWB33802*. Whereas, *QLr.cau-6DL* originates from the Chinese winter wheat landrace BQM without any exotic germplasm and consistently confers high resistance in the field and greenhouse tests. Thus, *QLr.cau-6DL* in BQM appears to be different from those QTL. Although further study will be required to determine the relationships between these loci, *QLr.cau-6DL* likely represents a novel QTL for reducing leaf rust severity.

RL6058 carries *Lr34* and the RL6058 × BQM population was segregating for *QLr.cau-6DL* and *Lr34*, which provided an opportunity for the comparison and combination between these two QTL. *Lr34* is known to be a major QTL for leaf rust resistance that is more effective than most QTL (Krattinger et al., 2009; Lagudah, 2011); it thus provides a baseline for evaluating the effect size of *QLr.cau-6DL*. Selection for *QLr.cau-6DL* based on cfd188 was as effective as selection for *Lr34* based on *cssfr5*. The MAS divided the RL6058 × BQM F_2_ seedlings into different groups (Figures 4, 5). The comparison in disease severity between groups indicates that *QLr.cau-6DL* could reduce leaf rust severity to a greater degree than *Lr34* and that it acted synergistically with *Lr34* to confer strong leaf rust resistance. An individual QTL generally provides insufficient protection under severe *Pt* epidemics; thus, pyramiding QTL has been considered as a strategy to enhance resistance. Herrera-Foessel et al. (2012) described an example where the combined effect of three slow rusting genes *Lr34*, *Lr46*, and *Lr68* resulted in near immunity, even though these genes pleiotropically induced leaf tip necrosis. BQM, the donor of *QLr.cau-6DL*, showed no leaf tip necrosis. Hence, *QLr.cau-6DL* may have some defense mechanism different from that of *Lr34*, *Lr46*, and *Lr68*. The combination of *QLr.cau-6DL* and *Lr34* should enhance the genetic diversity of leaf rust resistance.

The *QLr.cau-6DL* was consistently effective across all the trials involving four wheat crosses (NG × BQM, RL6058 × BQM, AK58 × BQM, and JM22 × BQM), three test locations (fields in Gansu and Shandong Provinces, and greenhouse conditions), and four crop seasons. The genetic backgrounds of the parental lines of the mapping and validating populations are diverse. NG is an American winter wheat cultivar, and RL6058 is a spring wheat from North America. AK58 and JM22 are the Chinese wheat commercial cultivars. The experiments were conducted in diverse environments (location × season). For instance, the experimental fields in Gansu (34°42′15″N, 104°40′08″E) and Shandong (36°18′09″N, 117°13′05″E) Provinces are geographically separated by a distance of more than 1,100 km and differ in elevation (1,950 vs. 90 m), annual average temperature (7.8 vs. 12.9°C), and annual precipitation (538.4 vs. 750.4 mm). *QLr.cau-6DL* showed consistent effects across the diverse genetic backgrounds and environments, indicating that it might have high value for breeding programs.

The APR in BQM, which is predominately conferred by *QLr.cau-6DL*, might be effective against many *Pt* races besides the artificially inoculated races THTT and FHTR, which were collectively virulent to the 31 designated *Lr* genes/alleles (*Lr1*, *Lr2a*, *Lr2b*, *Lr2c*, *Lr3*, *Lr3bg*, *Lr3ka*, *Lr10*, *Lr11*, *Lr14a*, *Lr14b*, *Lr15*, *Lr16*, *Lr17a*, *Lr18*, *Lr20*, *Lr21*, *Lr23*, *Lr25*, *Lr26*, *Lr28*, *Lr29*, *Lr30*, *Lr32*, *Lr33*, *Lr36*, *Lr39*, *Lr42*, *Lr45*, *Lr50*, and *LrB*) (Li et al., 2010). BQM showed APR under natural *Pt* infections from 1987 to 2020 in the field nursery in the southern Gansu Province, which features an environment conducive to the spread of leaf rust. During this period, *Pt* population were considerably diverse in terms of virulence variants. For example, seven *Pt* races were identified from only 30 samples collected from Gansu plots in 2014 (Du et al., 2015), and eleven *Pt* races were identified from 40 samples from Shandong plots in 2018 (Zhang et al., 2021), which is much fewer than the actual number of naturally occurring races due to the limited number of samples analyzed. Comprehensive surveys of *Pt* races have been performed over the wheat-growing areas of China by other researchers. Liu and Chen (2012) detected 79 races from 613 single-uredinial isolates collected between 2000 and 2006 from the 16 provinces, such as Gansu and Shandong. Ma et al. (2020) reported that *Pt* populations from the 5 provinces including Gansu exhibited high genetic diversity from 2013 to 2015 and accounted for 87.45% of the total observed genetic variation. BQM might have been subjected to infections by diverse *Pt* races and its APR was effective to these races. From 1987 to 2020, the disease severities of BQM were always lower than 25%, whereas the susceptible check MX had a severity of approximately 93% in the same field nursery.

For practical breeding, we introduced *QLr.cau-6DL* into AK58 and JM22 *via* backcrossing procedures (Supplementary Figure 1). AK58 and JM22 are elite Chinese wheat commercial cultivars with high yield and wide adaptability (Chen et al., 2016; Zhang et al., 2016), and they are widely used as parental lines in wheat breeding programs. The introgression of *QLr.cau-6DL* into AK58 and JM22 established a bridge for *QLr.cau-6DL* to be further transferred into new cultivars. Further work is underway to genotype other Chinese landraces or modern cultivars with the marker cfd188 for determining the frequency of *QLr.cau-6DL* and to fine-map *QLr.cau-6DL*.

## Conclusion

The results of our study indicate that the Chinese wheat landrace BQM confers APR to leaf rust for 34 years and reduces disease severity without triggering a hypersensitive response. Using a high-density genetic map and multiple field tests for QTL mapping, we identified a major QTL (*QLr.cau-6DL*) on chromosome 6DL from BQM. *QLr.cau-6DL* is likely a novel QTL with an effect size comparable to *Lr34*; it consistently reduced the leaf rust severity across different genetic backgrounds and diverse environments and was effective against various *Pt* races. The combination of *QLr.cau-6DL* with *Lr34*, based on the selection of markers cfd188 and *csffr5*, yielded a high level of resistance. The resistant germplasm and detected QTL could be potentially helpful to increase the genetic diversity of slow leaf-rusting resistance in wheat cultivars in breeding programs.

## Data Availability Statement

The datasets in the study are included in the article/Supplementary Material, further inquiries can be directed to the corresponding author.

## Author Contributions

MC and ZZ designed and conducted the experiments. ZZ and JF provided the study resources. MC, ZW, XJ, YZ, ZD, WQ, JR, and ZZ performed disease tests and the genotyping work. ZW constructed the chromosome linkage map. ZW, MC, and XJ analyzed disease data. ZW and MC drafted the manuscript. All authors contributed to the submitted manuscript.

## Conflict of Interest

The authors declare that the research was conducted in the absence of any commercial or financial relationships that could be construed as a potential conflict of interest.

## Publisher’s Note

All claims expressed in this article are solely those of the authors and do not necessarily represent those of their affiliated organizations, or those of the publisher, the editors and the reviewers. Any product that may be evaluated in this article, or claim that may be made by its manufacturer, is not guaranteed or endorsed by the publisher.

## Footnotes

1
http://www.capitalbiotech.com

2
http://www.illumina.com

3
http://www.isbreeding.net

4
http://www.kyazma.nl

5
http://mstmap.org/

6
http://wheatpedigree.net/
