# Supplementary material for: Identification and Validation of a Major Quantitative Trait Locus for Adult Plant Resistance Against Leaf Rust From the Chinese Wheat Landrace Bai Qimai
Source: Front Plant Sci. 2022 May 18;13:812002. doi: 10.3389/fpls.2022.812002 (PMC9158542; doi:10.3389/fpls.2022.812002)
Supplement: Supplementary file 7 [file Image_1.pdf]

Development of three validating wheat populations

RL6058 was crossed with BQM in May 2015 using BQM as pollen donor. In mid-October 2016, 700 F_2 seeds were sown in 5-mL centrifuge tubes filled with moist soil with a single germinated seed in each tube, and maintained in growth chambers at 5 ± 2 ℃ with a relative humidity higher than 70%. The DNA samples extracted from these seedlings were subjected to a selection based on the marker cfd188 for *QLr.cau-6DL* and the DNA marker *cssfr5* for *Lr34*. This selection resulted in four groups of QTL combination, namely, the F_2 seedlings with positive states for both cfd188 and *cssfr5* (representing *QLr.cau-6DL* + *Lr34*), positive states for cfd188 and negative states for *cssfr5* (*QLr.cau-6DL*), negative states for cfd188 and positive states for *cssfr5* (*Lr34*), and negative states for both cfd188 and *cssfr5* (None). From each of the four QTL combination groups, 20 F_2 seedlings were kept to the adult plant growth stage and they were self-pollinated in mid-May 2017 to produce $F_{2:3}$ family seeds.

Likewise, in mid-October 2016, 100 BC_3F_2 seeds derived from the cross AK58 × BQM (Figure S1) were sown in 5-mL centrifuge tubes, and DNA samples from these seedlings were selected for *QLr.cau-6DL* based on the marker cfd188. Five seedlings that were positive for cfd188 (R-group) and another five seedlings that were negative for cfd188 (S-group) were kept to the adult plant growth stage and they were self-pollinated in mid-May 2017 to produce $BC_3F_{2:3}$ family seeds. In the same way, R-group and S-group $BC_3F_{2:3}$ family seeds were obtained from the cross JM22 × BQM (Figure S1).

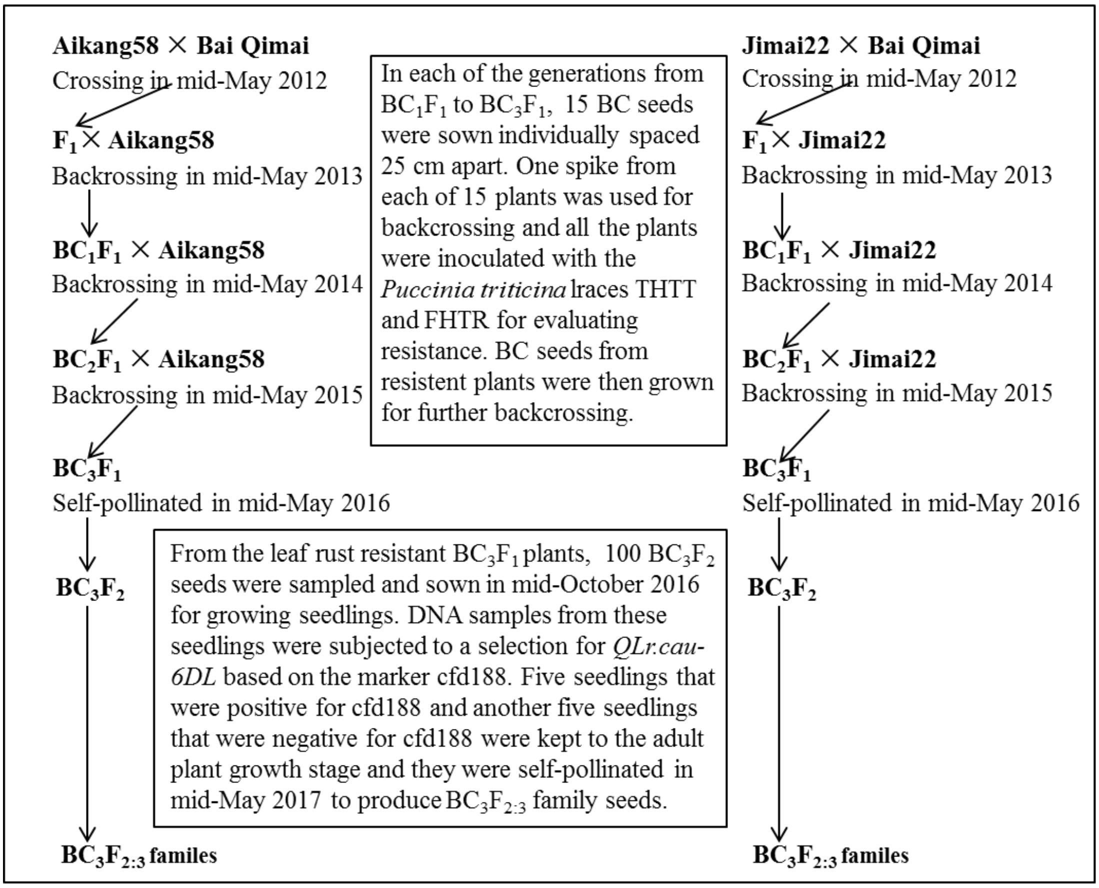

Figure S1. Development of two backcross populations.
